# Supplementary material for: Direct and indirect punishment of norm violations in daily life
Source: Nat Commun. 2020 Jul 9;11:3432. doi: 10.1038/s41467-020-17286-2 (PMC7347610; doi:10.1038/s41467-020-17286-2)
Supplement: Supplementary file 1 — Supplementary Information [file 41467_2020_17286_MOESM1_ESM.pdf]

Supplementary Information for

Direct and Indirect Punishment of Norm Violations in Daily Life

Catherine Molho [1,2]*

Joshua M. Tybur [1]

Paul A.M. Van Lange [1]

Daniel Balliet [1]

Affiliations

[1] VU Amsterdam, Department of Experimental and Applied Psychology, Institute for Brain and Behavior Amsterdam (IBBA), Van der Boechorststraat 7, 1081BT Amsterdam, the Netherlands

[2] Institute for Advanced Study in Toulouse, Esplanade de l'Université 1, 31080 Toulouse, Cedex 06, France

* Correspondence should be addressed to: catherine.molho@iast.fr

Supplementary Methods

Frequencies of Punishment

Daily punishment motivations. We ran a linear mixed model with punishment type (physical confrontation, verbal confrontation, gossip, and social exclusion), participant gender, and the participant gender × punishment type interaction predicting motivations to punish offenders. Across all daily reports of norm violations (k = 1,236), participants differentially endorsed motivations to engage in various types of punishment, $F(3, 2537.27) = 111.19$, $p < .001$. Bonferroni-corrected, pairwise comparisons are presented in Supplementary Table 1. Participants endorsed weaker motivations to punish via physical confrontation, compared with motivations to engage in all other types of punishment (all ps < .001). Further, motivations to punish via verbal confrontation were weaker compared to motivations to gossip about and exclude offenders (both ps < .001). There was no difference in the endorsement of motivations to gossip and socially exclude offenders. Finally, the overall effect of gender was non-significant ($F(1, 1169.76) = 0.07$, $p = .786$), and it was not moderated by punishment type ($F(3, 2537.27) = 0.35$, $p = .792$).

Daily punishment behaviors. We then used Generalized Estimating Equations (GEEs) with punishment type (confrontation, gossip, and social avoidance), participant gender, and the participant gender × punishment type interaction predicting punishment behaviors. Across all daily reports of norm violations (k = 1236), participants differentially engaged in various types of punishment behaviors, Wald $\chi^2(2) = 27.64$, $p < .001$. Specifically, they were more likely to gossip about offenders, rather than to directly confront (Wald $\chi^2(1) = 11.37$, $OR = 1.33$, $p = .001$) and socially avoid them (Wald $\chi^2(1) = 24.65$, $OR = 1.45$, $p < .001$). There was no main effect of participant gender (Wald $\chi^2(1) = 0.26$, $p = .612$), but there was a participant gender × punishment type interaction (Wald $\chi^2(2) = 8.08$, $p = .018$). Overall, women were less likely than men to directly confront offenders (Wald $\chi^2(1) = 7.37$, $b = -0.33$, $p = .007$). Planned contrasts showed

that, compared to direct confrontation, women were more likely to use gossip (Wald $\chi^2(1) = 7.47$, *OR* = 1.13, *p* = .006) and social avoidance (Wald $\chi^2(1) = 5.46$, *OR* = 1.09, *p* = .019).

We re-ran analyses only for events in which participants were present (*k* = 879). As above, participants differentially engaged in various types of punishment behaviors, Wald $\chi^2(2) = 12.63$, *p* = .002. There was no difference in the odds of directly confronting offenders or gossiping about them (Wald $\chi^2(1) = 0.20$, *p* = .658). Instead, both gossip (Wald $\chi^2(1) = 12.07$, *OR* = 1.37, *p* = .001) and direct confrontation (Wald $\chi^2(1) = 5.61$, *OR* = 1.31, *p* = .018) were more likely than avoidance of offenders.

Follow-up punishment behaviors. We used GEEs with punishment type (confrontation, gossip, and social avoidance), participant gender, and the participant gender × punishment type interaction predicting punishment behaviors in the follow-ups. Consistent with the overall pattern observed in daily assessments, participants differentially engaged in various types of punishment behaviors (*k* = 929), Wald $\chi^2(2) = 145.81$, $p < .001$. Gossip (in 45.4% of events) was more likely than confrontation (in 24.9% of events; Wald $\chi^2(2) = 102.47$, *OR* = 2.53, $p < .001$) and avoidance (in 27.9 % of events; Wald $\chi^2(1) = 73.11$, *OR* = 2.18, $p < .001$). The effect of gender was non-significant (Wald $\chi^2(1) = 0.57$, *p* = .448), and it was not moderated by punishment type (Wald $\chi^2(2) = 0.41$, *p* = .812).

In sum, participants endorsed stronger motivations to gossip about and socially exclude offenders compared to physically or verbally confronting them. Consistently, gossip was the most frequent behavioral response to norm violations in daily life, but direct confrontation was just as likely when immediate intervention was possible (i.e., when participants were physically present at the time of the violation). When focusing on violations in which participants were present, both direct confrontation and gossip were more prevalent than social avoidance.

Situations resembling second- and third-party punishment games. Our study used an inclusive definition of punishment—encompassing physical attacks, verbal confrontation, reputation manipulation, and withdrawal of benefits—and examined punishment in a broad range of situations (e.g., social dilemmas, coordination problems, and conventional norm violations) across various types of relationships. As such, the situations we considered in our study deviate from those included in experimental studies on cooperation, which typically focus on: (a) self-relevant offenses in which the perpetrators are strangers (i.e., second-party punishment situations), or other-relevant offenses in which both perpetrators and victims are strangers (i.e., third-party punishment situations).

In what follows, we report patterns of punishment behaviors in situations that more closely align with laboratory approaches to assessing second- and third-party punishment situations. We used GEEs with punishment type (confrontation, gossip, and social avoidance), participant gender, and the participant gender × punishment type interaction predicting punishment behaviors, only in those daily life situations that resemble second- and third-party punishment tasks.

When focusing on situations that more closely map onto second-party punishment paradigms used in the laboratory (i.e., self-relevant offenses in which the perpetrators are strangers; k = 403), we observed that participants differentially engaged in distinct types of punishment behaviors, Wald $\chi^2(2) = 14.73$, $p < .001$. Both gossip (in 48.9% of events; Wald $\chi^2(1) = 13.92$, $OR = 1.72$, $p < .001$) and social avoidance (in 47.4% of events; Wald $\chi^2(1) = 9.23$, $OR = 1.61$, $p = .002$) were more prevalent than direct confrontation (in 34.2% of events). Note that the rate at which direct confrontation occurred in these situations was similar to the rate of confrontation in the overall sample. The effect of gender was non-significant (Wald $\chi^2(1) = 0.51$, $p = .476$), and it was not moderated by punishment type (Wald $\chi^2(2) = 2.90$, $p = .234$).

When focusing on situations that more closely map onto third-party punishment paradigms used in the laboratory (other-relevant offenses that are committed by strangers *and* victimize strangers; k = 136), we again observed that participants differentially engaged in distinct types of punishment behaviors, Wald $\chi^2(2) = 23.15$, $p < .001$. Both gossip (in 39.0% of events; Wald $\chi^2(1) = 22.93$, $OR = 4.66$, $p < .001$) and social avoidance (in 36.0% of events; Wald $\chi^2(1) = 15.71$, OR = 4.14, $p < .001$) were more prevalent than direct confrontation (in 11.8% of events). Note that the rate at which direct confrontation occurred in these situations was much lower than that in the overall sample, suggesting that costly confrontation is rare in situations that resemble third-party punishment tasks[20,21,43]. The effect of gender was non-significant (Wald $\chi^2(1) = 0.22$, $p = .637$), and was not moderated by punishment type (Wald $\chi^2(2) = 0.04$, $p = .979$).

Welfare Tradeoff Ratios and Punishment

Welfare tradeoff ratios and daily punishment motivations. We ran a linear mixed model with punishment type (physical confrontation, verbal confrontation, gossip, and social exclusion), participant gender, WTR_{own}, and the participant gender × punishment type and WTR_{own} × punishment type interactions as predictors of punishment motivations.

Within-person changes in WTR toward offenders were overall negatively associated with motivations to engage in punishment, $F(1, 697.57) = 15.69$, $p < .001$. Consistent with predictions, there was a WTR_{own} (within-person centered) × punishment type interaction predicting motivations to engage in punishment, $F(3, 4253.25) = 19.92$, $p < .001$. Within-person changes in WTR_{own} were more strongly, negatively associated with social exclusion compared with motivations to engage in all other types of punishment (all ps $\leq .001$; Supplementary Table 2).

We observed a similar pattern of results for the relations between person-average WTR_{own} and motivations to punish offenders. People who generally valued offenders more reported weaker punishment motivations overall, $F(1, 922.13) = 26.37$, $p < .001$. Further, there was a

WTR_{own} (person-average) × punishment type interaction predicting motivations to punish offenders, $F(3, 4433.33) = 21.65$, $p < .001$. People who generally valued offenders more endorsed weaker motivations to socially exclude them, compared with motivations to physically and verbally confront them (both *p*s < .001). Person-average WTR_{own} did not differentially relate with motivations to gossip about or socially exclude offenders ($p = .987$; see Supplementary Table 2).

Additionally, we wanted to test the possibility that participants' motivations to punish offenders also vary depending on their perceptions of how much offenders value them—i.e., WTR_{other}. To do so, we ran another linear mixed model with punishment type (physical confrontation, verbal confrontation, gossip, and social exclusion), participant gender, WTR_{own}, WTR_{other}, and the participant gender × punishment type, WTR_{own} × punishment type, and WTR_{other} × punishment type interactions as predictors of punishment motivations.

When including WTR_{other} and the WTR_{other} × punishment type interaction in the model, the pattern of relations between WTR_{own} and punishment motivations remained virtually the same as reported above (see also Supplementary Table 2). The effects of both within-person and person-average WTR_{other} on punishment motivations were non-significant (both *p*s > .09) and were not moderated by punishment type (both *p*s > .07).

In sum, both within-person and person-average changes in WTR toward offenders (i.e., WTR_{own}) were negatively associated with participants' motivations to engage in punishment. More pertinent to our hypotheses, when people valued offenders more, either in a specific situation or across situations, they endorsed motivations to punish via indirect means less so than motivations to punish via confrontational means. Situational and person-average changes in perceptions of offenders' WTR toward oneself (i.e., WTR_{other}) were not associated with participants' motivations to punish offenders.

Welfare tradeoff ratios and daily punishment behaviors. We used GEEs with punishment type (confrontation, gossip, and social avoidance), participant gender, WTR_{own}, and the participant gender × punishment type and WTR_{own} × punishment type interactions as predictors of punishment behaviors.

Overall, within-person changes in WTR_{own} were not associated with the odds of engaging in punishment (Wald $\chi^2(1) = 2.73$, $p = .098$). More pertinent to our hypotheses, though, there was a WTR_{own} (within-person centered) × punishment type interaction predicting punishment behaviors, Wald $\chi^2(2) = 74.42$, $p < .001$. Planned contrasts indicated that situational increases in WTR toward offenders were associated with increased odds of direct confrontation compared to both gossip (Wald $\chi^2(1) = 47.56$, $OR = 1.28$, $p < .001$) and social avoidance (Wald $\chi^2(1) = 68.95$, $OR = 1.35$, $p < .001$).

Person-average changes in WTR toward offenders were overall negatively associated with the odds of engaging in punishment, Wald $\chi^2(1) = 5.55$, $p = .018$. There was also a WTR_{own} (person-average) × punishment type interaction predicting punishment behaviors, Wald $\chi^2(2) = 41.14$, $p < .001$. As above, planned contrasts indicated that higher person-average WTR_{own} was associated with higher odds of direct confrontation compared to gossip (Wald $\chi^2(1) = 25.34$, $OR = 1.17$, $p < .001$) and compared to social avoidance (Wald $\chi^2(1) = 37.84$, $OR = 1.25$, $p < .001$).

As earlier, we wanted to test the possibility that participants' punishment behaviors may also vary depending on their perceptions of the value that offenders place on their welfare (WTR_{other}). To do so, we ran another linear mixed model with punishment type (confrontation, gossip, and social avoidance), participant gender, WTR_{own}, WTR_{other}, and the participant gender × punishment type, WTR_{own} × punishment type, and WTR_{other} × punishment type interactions as predictors of punishment behaviors.

When including WTR_{other} and the WTR_{other} × punishment type interaction in the model, the pattern of relations between WTR_{other} and punishment behaviors remained virtually unchanged. The effects of within-person and person-average WTR_{other} on punishment behaviors were non-significant (both *p*s > .07). However, we observed a WTR_{other} (person-centered) × punishment type interaction predicting punishment behaviors, Wald $\chi^2(2) = 9.27$, $p = .010$. Planned contrasts indicated that situational increases in offenders' WTR toward oneself were associated with higher odds of direct confrontation compared to both gossip (Wald $\chi^2(1) = 4.28$, $OR = 1.08$, $p = .039$) and social avoidance (Wald $\chi^2(1) = 9.09$, $OR = 1.14$, $p = .003$). There was no WTR_{other} (person-average) × punishment type interaction predicting punishment behaviors (Wald $\chi^2(2) = 4.11$, $p = .128$).

In sum, both within-person, situational changes and person-average changes in WTR toward offenders were differentially associated with the odds of distinct punishment behaviors. When people valued offenders more, either in a specific situation or across situations, they were more likely to directly confront them compared to gossiping about or socially avoiding them. Further, within-person, situational changes in perceptions of offenders' WTR toward oneself were differentially associated with the odds of distinct punishment behaviors. In situations in which participants perceived offenders as valuing them more highly, they were more likely to directly confront offenders rather than use indirect means to punish them.

Victim of Offenses and Punishment

Victim of offenses and daily punishment motivations. We ran a linear mixed model with punishment type (physical confrontation, verbal confrontation, gossip, and social exclusion), participant gender, and a variable coding the victim of offenses (1 = self; 2 = other) as predictors of motivations to engage in various types of punishment. The model also included the participant gender × punishment type and victim × punishment type interactions. The endorsed punishment

motivations were not predicted by the victim of offenses (F(1, 1713.11) = 0.32, p = .571), or the victim × punishment type interaction (F(3, 2640, 35) = 1.66, p = .174).

Victim of offenses and daily punishment behaviors. We used GEEs with punishment type (confrontation, gossip, and social avoidance), participant gender, victim, and the participant gender × punishment type and victim × punishment type interactions as predictors of punishment behaviors.

Results showed that being personally victimized by a violation (compared to someone else being victimized) was positively associated with punishment, Wald $\chi^2(1) = 94.23$, $p < .001$. In response to self-relevant (compared to other-relevant) violations, participants were more likely to directly confront (Wald $\chi^2(1) = 84.98$, $b = 1.07$, $p < .001$), gossip about (Wald $\chi^2(1) = 19.13$, $b = 0.45$, $p < .001$) *and* socially exclude offenders (Wald $\chi^2(1) = 14.81$, $b = 0.42$, $p < .001$). Importantly, we observed a victim × punishment type interaction, Wald $\chi^2(2) = 19.97$, $p < .001$, such that being personally victimized by a violation had a stronger effect on direct confrontation, compared to gossip and avoidance (both ps < .001; see main text).

In sum, participants' endorsement of motivations to punish norm violations was unrelated to whether they were personally victimized or whether they were mere observers. However, self-relevant violations were associated with increased odds of all punishment behaviors, especially with direct confrontation of offenders.

Moral Wrongness and Punishment

Moral wrongness and daily punishment motivations. We ran a linear mixed model with punishment type (physical confrontation, verbal confrontation, gossip, and social exclusion), participant gender, moral wrongness, and the participant gender × punishment type and moral wrongness × punishment type interactions as predictors of motivations to engage in various types of punishment.

Within-person, situational increases in moral wrongness were positively associated with endorsement of motivations to punish offenders, $F(1, 1471.93) = 120.06$, $p < .001$. Further, there was a moral wrongness (within-person centered) × punishment type interaction, $F(3, 4008.72) = 3.92$, $p = .008$. Planned contrasts indicated that moral wrongness (within-person centered) was most strongly, positively associated with motivations to socially exclude offenders ($b = 0.51$, $p < .001$), compared with motivations to engage in all other types of punishment (all *p*s <.01, see Supplementary Table 3).

We observed similar patterns for the relations between person-average moral wrongness and motivations to punish offenders. People who in general perceived violations as more morally wrong endorsed stronger motivations to punish offenders overall, $F(1, 1306.88) = 62.39$, $p < .001$. Further, there was a moral wrongness (person-average) × punishment type interaction, $F(3, 4213.49) = 4.19$, $p = .006$. Moral wrongness (person-average) was most strongly associated with motivations to socially exclude offenders ($b = 0.42$, $p < .001$; see Supplementary Table 3).

In sum, both within-person, situational increases in moral wrongness and person-average increases in moral wrongness predicted greater motivations to engage in various types of punishment. These effects were stronger for motivations to socially exclude offenders compared with motivations to engage in other types of punishment.

Moral wrongness and daily punishment behaviors. We used GEEs with punishment type (confrontation, gossip, and social avoidance), participant gender, moral wrongness, and the participant gender × punishment type and moral wrongness × punishment type interactions as predictors of punishment behaviors.

Within-person increases in moral wrongness were overall positively associated with the odds of engaging in punishment, Wald $\chi^2(1) = 5.93$, $p = .015$. Further, there was a moral wrongness (within-person centered) × punishment type interaction predicting punishment

behaviors, Wald $\chi^2(2) = 20.62$, $p < .001$. Planned contrasts indicated that moral wrongness (within-person centered) was associated with lower odds of direct confrontation compared to both gossip (Wald $\chi^2(1) = 15.76$, $OR = 0.68$, $p < .001$) and to social avoidance (Wald $\chi^2(1) = 16.15$, $OR = 0.67$, $p < .001$).

However, we observed no association between person-average moral wrongness and the odds of punishment. People who in general perceived violations as more morally wrong were not more likely to engage in punishment behaviors (Wald $\chi^2(1) = 0.30$, $p = .585$). There was also no moral wrongness (person-average) × punishment type interaction predicting punishment behaviors (Wald $\chi^2(2) = 4.24$, $p = .120$).

In sum, within-person, situational increases in moral wrongness predicted decreased odds of directly confronting offenders, but increased odds of gossip and social avoidance. Person-average changes in moral wrongness were not associated with different punishment behaviors.

Perceived Power and Punishment

Perceived power and daily punishment motivations. We ran a linear mixed model with punishment type (physical confrontation, verbal confrontation, gossip, and social exclusion), participant gender, power, and the participant gender × punishment type and power × punishment type interactions as predictors of punishment motivations.

Within-person changes in perceived power were negatively associated with motivations to punish offenders, $F(1, 1635.80) = 7.18$, $p = .007$. Further, there was a perceived power (within-person centered) × punishment type interaction, $F(3, 4247.63) = 2.75$, $p = .041$. This interaction was driven by power more strongly relating to motivations to socially exclude compared to verbally confront offenders ($p = .011$) (see Supplementary Table 4).

We observed similar patterns for the associations between person-average power and motivations to punish offenders. People who in general perceived themselves to lack power had

greater punishment motivations, $F(1, 1292.24) = 18.51$, $p < .001$. Further, there was a power (person-average) × punishment type interaction predicting motivations to engage in various types of punishment, $F(3, 4114.19) = 3.36$, $p = .018$. The interaction was driven by power more strongly, negatively relating with motivations to socially exclude offenders compared to motivations to physically confront offenders ($p = .032$) (see Supplementary Table 4).

In sum, lower power predicted higher endorsement of motivations to punish offenders, and this effect was stronger for motivations to socially exclude offenders compared with motivations to engage in confrontational punishment.

Perceived power and daily punishment behaviors. We used GEEs with punishment type (confrontation, gossip, and social avoidance), participant gender, power, and the participant gender × punishment type and power × punishment type interactions as predictors of punishment behaviors.

Overall, within-person changes in perceived power were not associated with the odds of engaging in punishment behaviors (Wald $\chi^2(1) = 0.97$, $p = .324$). More relevant to our hypotheses, though, there was a power (within-person centered) × punishment type interaction predicting punishment behaviors, Wald $\chi^2(2) = 42.19$, $p < .001$. Planned contrasts indicated that situational increases in power were associated with higher odds of direct confrontation compared to gossip (Wald $\chi^2(1) = 36.78$, $OR = 1.91$, $p < .001$) and to social avoidance (Wald $\chi^2(1) = 32.24$, $OR = 1.89$, $p < .001$).

Further, there was no association between person-average power and punishment behaviors (Wald $\chi^2(1) = 0.80$, $p = .370$). However, we observed a power (person-average) × punishment type interaction predicting punishment behaviors, Wald $\chi^2(2) = 11.49$, $p = .003$. People who generally perceived themselves as having more power were more likely to directly

confront offenders compared to gossiping about them (Wald $\chi^2(1) = 11.41$, *OR* = 1.57, *p* = .001) and compared to socially avoiding them (*OR* = 3.39, *p* = .065).

In sum, both within-person, situational increases and person-average increases in power differentially related to punishment behaviors. Participants with more power, either in a specific situation or across situations, were more likely to directly confront offenders rather than gossip about them or socially avoid them.

Emotions and Punishment

Anger, disgust, and daily punishment motivations. We ran a linear mixed model with punishment type (physical confrontation, verbal confrontation, gossip, and social exclusion), participant gender, anger and disgust as predictors of motivations to engage in punishment. The model also included the participant gender × punishment type, anger × punishment type, and disgust × punishment type interactions as predictors.

Within-person increases in anger ($F(1, 372.55) = 67.16$, $p < .001$) and disgust ($F(1, 450.90) = 42.18$, $p < .001$) were associated with stronger motivations to engage in punishment. Further, there was a disgust (within-person centered) × punishment type interaction, $F(3, 4166.67) = 3.79$, $p = .010$. When participants felt more disgust, they reported stronger motivations to gossip about and socially exclude offenders, compared to physically confronting them. Disgust was not differentially related with motivations to physically and verbally confront offenders (see Supplementary Table 5). Further, there was no anger (within-person centered) × punishment type interaction, $F(3, 4277.69) = 2.17$, $p = .089$.

We observed similar patterns for the associations between person-average anger and disgust and motivations to engage in punishment. People who generally experienced more anger ($F(1, 1309.93) = 36.45$, $p < .001$) and disgust ($F(1, 1429.56) = 35.78$, $p < .001$) had greater motivations to engage in various types of punishment. There was also a disgust (person-average)

× punishment type interaction, $F(3, 4439.01) = 6.81$, $p < .001$. Participants who on average felt more disgust toward offenders had greater motivations to verbally confront, gossip about, or socially exclude them, compared to physically confronting them (see Supplementary Table 5). There was also an anger (person-average) × punishment type interaction, $F(3, 4262.32) = 3.04$, $p = .028$, driven by anger more strongly relating to motivations to verbally confront offenders rather than physically confront them.

In sum, we observed that within-person, situational increases, as well as person-average increases, in anger and disgust were positively associated with endorsed motivations to punish offenders. Further, increases in felt disgust, both in specific situations and across situations, were more strongly associated with motivations to punish offenders via indirect means compared with motivations to punish them via confrontational means.

Anger, disgust, and daily punishment behaviors. We used GEEs with punishment type (confrontation, gossip, and social avoidance), participant gender, anger, disgust, and the participant gender × punishment type, anger × punishment type, and disgust × punishment type interactions as predictors of punishment behaviors.

Overall, within-person increases in anger (Wald $\chi^2(1) = 7.62$, $p = .006$) and disgust (Wald $\chi^2(1) = 10.36$, $p = .001$) were positively associated with the odds of punishment. More relevant to our hypotheses, we observed that within-person changes in disgust were differentially associated with distinct behaviors, Wald $\chi^2(2) = 11.43$, $p = .003$. Specifically, increases in disgust were associated with higher odds of engaging in gossip (Wald $\chi^2(1) = 11.43$, $b = 0.28$, $p = .001$) and social avoidance (Wald $\chi^2(1) = 3.98$, $b = 0.18$, $p = .046$) compared to direct confrontation. Within-person changes in anger were not differentially associated with distinct behaviors (Wald $\chi^2(2) = 3.38$, $p = .185$).

We observed the same pattern for the associations between person-average emotions and punishment behaviors. Participants who on average experienced more disgust were more likely to engage in punishment (Wald $\chi^2(1) = 10.49$, $p = .001$), and person-average disgust had a stronger effect on gossip (Wald $\chi^2(1) = 11.02$, $b = 0.41$, $p = .001$) and social avoidance (Wald $\chi^2(1) = 16.65$, $b = 0.56$, $p < .001$) compared to direct confrontation (interaction effect: Wald $\chi^2(2) = 17.99$, $p < .001$). There were no effects of person-average anger on punishment behaviors (Wald $\chi^2(1) = 1.04$, $p = .307$; interaction effect: Wald $\chi^2(2) = 1.16$, $p = .560$).

In sum, both within-person, situational changes and person-average changes in disgust were differentially associated with punishment behaviors. Disgust, both in a specific situation and across situations, was more strongly, positively associated with indirect rather than direct punishment behaviors.

Additional Exploratory Analyses

Motivated by a reviewer's suggestions, we explored whether the observed patterns of associations between perceived power and punishment motivations and behaviors were altered when accounting for the closeness of participants' relationship to offenders.

Exploratory analyses – punishment motivations. We ran a linear mixed model with punishment type (physical confrontation, verbal confrontation, gossip, and social exclusion), participant gender, power, and the participant gender × punishment type and power × punishment type interactions as predictors of punishment motivations, while also controlling for participants' WTR toward offenders and the WTR × punishment type interaction.

When controlling for participants' WTR toward offenders, we observed no effects of within-person changes in power on motivations to punish offenders (within-person power × punishment type interaction, $F(3, 3966.73) = 1.15$, $p = .327$; main effect of within-person power, $F(1, 1099.42) = 2.60$, $p = .107$). Further, there was no interaction of power (person-average) ×

punishment type, $F(3, 4343.91) = 1.22$, $p = .301$. However, participants who generally perceived themselves to lack power reported greater motivations to engage in punishment, $F(1, 1171.07) = 15.01$, $p < .001$, consistent with the results of our main analyses.

We also ran a linear mixed model with punishment type (physical confrontation, verbal confrontation, gossip, and social exclusion), participant gender, power, and the participant gender × punishment type and power × punishment type interactions as predictors of motivations to punish offenders, while also controlling for participants' emotional closeness toward offenders and the emotional closeness × punishment type interaction.

When controlling for emotional closeness toward offenders, we observed no effects of within-person changes in power on motivations to punish offenders (within-person power × punishment type interaction, $F(3, 3993.13) = 1.34$, $p = .259$; main effect of within-person power, $F(1, 1275.64) = 0.84$, $p = .360$). Further, there was no interaction of power (person-average) × punishment type in predicting motivations to punish offenders, $F(3, 4398.81) = 0.85$, $p = .466$. Again, participants who generally perceived themselves to lack power reported greater motivations to engage in various types of punishment, $F(1, 1311.59) = 6.77$, $p = .009$.

In sum, when controlling for WTR toward offenders or emotional closeness, situational changes in power were not associated with motivations to engage in punishment. Those individuals who on average perceived themselves as having lower power endorsed overall stronger motivations to punish offenders.

Exploratory analyses – punishment behaviors. We used GEEs with punishment type (confrontation, gossip, and social avoidance), participant gender, power, and the participant gender × punishment type and power × punishment type interactions as predictors of punishment behaviors, while *also* controlling for participants' WTR toward offenders and the WTR × punishment type interaction.

When controlling for WTR toward offenders, within-person changes in power were not associated with the odds of engaging in punishment behaviors (Wald $\chi^2(1) = 1.84$, $p = .175$), but there was a power (within-person centered) × punishment type interaction predicting punishment behaviors (Wald $\chi^2(2) = 25.70$, $p < .001$). Situational increases in power were associated with higher odds of confrontation compared to gossip (Wald $\chi^2(1) = 23.17$, *OR* = 1.71, $p < .001$) and social avoidance (Wald $\chi^2(1) = 18.34$, *OR* = 1.65, $p < .001$). Consistent with our main analyses, there was no association between person-average power and punishment behaviors (Wald $\chi^2(1) = 0.11$, $p = .734$). However, we observed a power (person-average) × punishment type interaction predicting punishment behaviors (Wald $\chi^2(2) = 7.14$, $p = .028$). People who generally perceived themselves as having more power were more likely to confront offenders compared to gossiping about them (Wald $\chi^2(1) = 6.15$, *OR* = 1.42, $p = .013$), but not compared to avoiding them (Wald $\chi^2(1) = 0.56$, *OR* = 1.12, $p = .453$).

Finally, we used GEEs with punishment type (confrontation, gossip, and social avoidance), participant gender, power, and the participant gender × punishment type and power × punishment type interactions as predictors of punishment behaviors, while also controlling for participants' emotional closeness toward offenders and the emotional closeness × punishment type interaction.

When controlling for emotional closeness toward offenders, within-person changes in power were again not associated with punishment behaviors (Wald $\chi^2(1) = 1.55$, $p = .213$). There was a power (within-person centered) × punishment type interaction predicting punishment behaviors (Wald $\chi^2(2) = 26.44$, $p < .001$), such that increases in situational power were associated with higher odds of confrontation compared to gossip (Wald $\chi^2(1) = 23.91$, *OR* = 1.71, $p < .001$) and social avoidance (Wald $\chi^2(1) = 18.00$, *OR* = 1.64, $p < .001$). Further, there was no association between person-average power and punishment behaviors (Wald $\chi^2(1) = 3.58$, $p = .058$).

However, we observed a power (person-average) × punishment type interaction predicting punishment behaviors (Wald $\chi^2(2) = 7.98$, $p = .019$). People who generally perceived themselves as having more power were more likely to confront offenders, although the odds of confrontation were not statistically different from gossip ($p = .070$) or avoidance ($p = .483$).

In sum, when controlling for WTR toward offenders or emotional closeness, we observed similar patterns of results to those of our main analyses. Within-person, situational increases and person-average increases in power differentially related to punishment behaviors. Participants with more power in a specific situation were more likely to directly confront offenders rather than gossip about them.

Examples of Norm Violations.

Self-relevant norm violations:

1. Someone approached me at the train station and asked if I wanted to buy a (stolen bike).
2. A friend expressed himself negatively about Islam multiple times.
3. An acquaintance asked me if I wanted to take part in a photoshoot. She wasn’t very communicative but eventually we agreed on meeting on June the 26th at 1 o’clock in the afternoon. But she didn’t give me a location and haven’t heard from her since I asked for it.
4. A homeless person with whom I had a nice conversation, suddenly went crazy when the bus driver didn’t close the door quick enough and loudly screaming he banged against the bus door.
5. A patient in the hospital acted rudely toward me (doctor in training) and made condescending remarks.
6. I’m in a hotel and filled up my 2 bottles of water during the breakfast buffet. I heard a man at the table next to me say to his table companions that this wasn’t allowed.
7. The supervisor at work complained about the way tests were being done. He tried to influence the results.
8. Someone screamed at me when I wanted to help out.
9. The car driver crossed the red light.
10. I was working when a man without shirt came to ask things. Some other guests were looking a bit uncomfortable.
11. I was on a bike and a parked car drove past me to get onto the road. The driver saw I had to brake hard and shouted “boo” from the window.
12. Bus driver for a Texel taxi was very rude and didn’t help us by driving 5 minutes further, resulting in us having to walk half an hour with our luggage.

13. When I was parking this afternoon, some other driver deemed it necessary to honk loudly. He probably thought I wasn’t fast enough.

14. I was called with a question regarding my sick father. The callers were my fathers’ friends. The questions made me feel cornered.

15. He tried to flirt with me and I didn’t take it kindly.

16. He told me about his father’s finances, who just passed away. This was rather inappropriate.

17. Last week I bought pizzas and put them in the common freezer. One of my roommates took a pizza from the freezer. I found my pizza in the kitchen, defrosted.

18. We were having lunch with colleagues. One colleague, who I don’t really get, ridiculed clothing of other colleagues passing by. They didn’t hear it but it gave me an unpleasant feeling.

19. Went with my son to the swimming pool in the Vondelpark and saw a man making pictures from naked children at a distance (not my son, he was wearing something).

20. An order won’t go through because someone claims to have never agreed to it, even though he did. Perhaps his superior wasn’t aware and now he is shouting that he never agreed to it.

21. Perpetrator is a festival organizer. There were holes in the road, covered with sand, which made me fall over. jacket dirty, beer spilled over my watch and a customer service nowhere to be seen. bad.

22. Builders at the neighbors downstairs cut off the main water valve without informing anybody, even though they promised last week (when the same happened) to always give a heads up first.

23. Someone in the supermarket committed shoplifting. I don't know her and she's not from around here.

24. Mother of babysitting family was 45 min late.

25. The neighbors were in a fiery argument and I watched them from my balcony. It was a couple and they cursed at each other.

Other-relevant norm violations:

1. Impolite addicts did not want to pay for beer at the supermarket.

2. A boy at the service desk in the super market was all ruffled up because someone cursed him.

3. During News Hour, people from Groningen were speaking about having a hard time getting damage repairs from the Dutch Oil Company. According to the Dutch Oil Company, it keeps needing further investigation, while the people from Groningen suffer.

4. Tennis trainer reacted on a wrongly played ball. Player said he also knew that.

5. A friend of mine was lying on a nude beach and the perpetrator was looking at people with binoculars.

6. My partner wanted to be of too much help.

7. Client does not want his colleague to help him.

8. This is not something I experienced first-hand. Today was my day off and I have been inside all day. What bothered me was something I saw in the media. The immigration policy in the US, where parents are separated from their children. I find this disgusting and not right.

9. The tenant of a training area was creating a problem when our club trained there for the first time last week.

10. On Pinkpop, four people were hit by a car. One dead. Three heavily wounded. The perpetrator ran.

11. Someone told me that he was cheated on in a relationship.

12. Someone was being yelled to.

13. One of my uncles went on about refugees and that they should not have been taken in, yada yada

14. The same person yelled, was aggressive, verbally and physically.

15. Neighbor lets her dog poop in the yard. The whole neighborhood experiences the stench.

16. Car driver who wants to keep driving while the traffic worker points out that the road is closed.

17. In a one-way road, despite the numerous signs that portray this, still pull onto the wrong side of the road to not have to drive around. This happens structurally.

18. A perpetrator (passed away this week, was now 94), used to be very strict to his children, so the son became rebellious.

19. It was a person from Dutch descent (employee at a diner) who was being somehow patronizing to the person who spoke English. There was a very mean reaction to a simple question.

20. In the supermarket, I see a man suddenly turn his cart without looking to go the other way. He almost hits another customer who walked there. He starts to mumble, but still loudly, blaming the other customer.

21. Scooter store did not sign out a scooter, so we got a fine for driving without insurance, while we do not own the scooter for two years.

22. I saw two people getting into a fight at a cafe, due to a soccer game between Tunis and Belgium.

23. A customer in a store left his trash.

24. One of the students was very angry and rebellious because he is getting another teacher after the holiday, made ugly gestures to a teacher.

25. Same case. Colleague is being treated poorly by manager.

Examples of Punishment Responses.

Direct confrontation.

1. Sent an email expressing my discontent.

2. I explained to him that I thought it was annoying.

3. Asked her why she did it and how honest she was about it with her husband.

4. I yelled that he should give way to us and called him a fool.

5. I said that it's not really appropriate, but at that age, I might have done it as well.

6. I argued, I threatened, I listened, I have withdrawn my threat, I made peace.

7. I got mad at my boyfriend and told him that, next time, he should clean it up himself.

8. I immediately told him that I didn't like what he was doing and that he had to think about the person he was affecting. Karma stepped in immediately when the valve was not working properly and his tire broke.

9. I caught my partner in a lie and sent the proof of this to my partner. Then, I confronted him.

10. I confronted her with the situation and told her what things she could improve. Other than that she has to hand in her mobile phone as soon as we get home.

11. I spoke to him about his behavior and told him that I do not appreciate what he did. And that next time he should make sure he knows what actually happened before accusing someone.

12. I stopped the violator by shouting at her and fighting with her so I would out-argue her.

13. Made it clear that this behavior is anti-social.

14. I said that I did not agree with it. I said that she should have discussed with me about how we should handle it.

15. I angrily called my brother and told him that he had forgot to give me the cap back.

16. I snapped and said that the offender should know the rules better.

17. I was one of the referees, so I tried to step in as much as possible.

18. When he found the object, I reminded him about his exaggerated reaction.

19. I yelled loudly at the runner.

20. I got into an argument with my boyfriend.

21. I told him that he should no longer count on me. From now on, he can get his own help.

22. I screamed at her, causing her to see me and stop. I then confronted her with the fact that she had almost hit us by looking at her phone.

23. I said it was not okay that she threw away the can.

24. Confront together.

25. I called the group together and explained why this is unacceptable. I told the specific student that I will respond to it.

Gossip.

1. I gave some subtle hints but did not dare to say it directly since she has to grade me for my master’s internship of 9 months.

2. I have only shared my irritation with my partner, who heard it as well, but it did not bother him as much as me.

3. We actually laughed really hard. We hate the boss and hope that he cannot find a replacement for the entire team that resigned. It was sad for that woman, but perhaps she was saved from a lousy job.

4. I waited a little bit longer to see if it would stop on its own. In the meantime, I complained to my family. It stopped on its own.

5. I discussed the matter with my husband and friends. There´s nothing else you can do as an inhabitant of `Noord-Holland'. I cannot go protesting on the behalf of Groningen.

6. I listened to the story of my mother, astonished.

7. I shared this news with friends. But other than that there is not much I could do.

8. I complained to my co-driver and flashed my big lights to the violator to irritate him.

9. I read it in disbelief and talked with others about how awful this behavior is.

10. I later told someone else that I did not like the behavior.

11. I asked how the others felt about it.

12. My mom and I discussed about how we find this inappropriate.

13. I watched what went down. One of the employees from 'Zara' stepped in so I did not have to address anyone.

14. I gave the victims advice on how they could solve it in a peaceful manner.

15. I watched to be sure that the woman was not being abused physically, I was ready to intervene. When she drove away, I went inside.

16. I only heard about it afterwards, but I did not think it was very kind, but I would not express this to her.

17. Tried to let it go. At home, I told my husband about it.

18. I walked away and reported it to a colleague.

19. Shared this behavior with colleagues.

20. I heard about this via a colleague, who had heard about this within his network. I then passed this on to the management, they asked him for clarification.

21. Addressing the offenders was not an option, they had already left. We did however talk about it with 4 others. Our conclusion: unwanted behavior.

22. I did not confront him or avoid contact even though I found him dominantly present. In the future, it will be less easy for me to hang out with him.

23. I swallowed the comments and continued working. I did not feel like I was in the position to say something since it concerns a dependent relationship (it is my supervisor).

24. I did not do anything about it. A woman behind me said that it is not allowed to do this, but I indicated that this just happens and it is best not to make a big deal out of it.

25. This situation is quite common, so I don't react to it very often anymore. It struck me that there were people cutting in line. I have talked about this with others, but not with the victim and the person cutting in line.

Social avoidance.

1. I avoided him as much as possible.

2. My hands were full with bags and I did not feel flexible enough to show them a response in the form of bodily communication. I did not say anything and let it happen. Preferably I would have said that the line started in the back.

3. When the behavior took place, I continued a conversation with another colleague and said once "this is really not okay" and shook my head. After this I had no more contact with him, which wasn't necessary since we don't work together

4. I let the other teacher react, it did not feel right to get involved.

5. I changed the subject. This distracted him.

6. Ignore and do not make contact.

7. Nothing, I was too scared to say something. There was a handyman today and I am actually not sure if he can do anything for me.

8. I did nothing, except watch and listen. I stepped aside so I would not (accidentally) end up in the middle. I let the bouncer do his job.

9. Right now, since it just happened an hour ago, we're not speaking. She thinks she has been wronged and I will not admit to that. I think she's mature enough to solve problems on her own.

10. I became more quiet.

11. I thought to myself: let it go, because I just know this person is weird.

12. I said nothing because there were a lot of others around. Furthermore I avoided social contact.

13. I stepped aside and stayed out of it.

14. I ignored my partner.

15. I changed the schedule at my own expense. I did not address her about her (for me inappropriately detrimental) behavior, and avoided further social interaction.

16. I did not do anything but I did get annoyed.

17. As I have enough other stuff to worry about I did not do anything about this situation…yet. But I will do so in the future. They do not know that it is bothering me so I will have to talk to them about it. But not with him, since that could be dangerous for me. Or maybe I can talk to him, if I do it in a jokey way. I will talk to the manager...that will be the best...I think.

18. I ignored him because our child was present and I did not want the situation to get worse.

19. Nothing, I just do not want to talk to him anymore.

20. I did nothing. I gave a disapproving look but this went unnoticed as they were too busy with each other.

21. I kept talking to him the way I normally would. Whenever he touched me I acted as if it did not happen. I continued laughing and talking.

22. I walked away with my friend.

23. There were others present. They were there sooner than me. I saw that someone intervened.

24. I said that it was meant as a joke but that I will not send videos to him anymore.

25. I tried to ignore the man. Eventually I went home.

Supplementary Tables

Supplementary Table 1. Endorsement of distinct punishment motivations

Reference Category	Physical	Verbal	Gossip
Verbal	-0.31 (.037) ***	-	
Gossip	-0.69 (.039) ***	-0.37 (.037) ***	-
Social Exclusion	-0.66 (.039) ***	-0.34 (.039) ***	-0.03 (.037)

Bonferroni-corrected pairwise comparisons of endorsement of distinct motivations. Asterisks indicate *p* values < .001. The first column shows the reference category. Standard errors are provided in parentheses. Source data are provided as a Source Data file.

Supplementary Table 2. Valuation of offenders and endorsement of punishment motivations

	Estimate	*t*	*df*	*p*
Intercept	3.11	57.51	3473.32	< .001
Physical	-0.96	-15.33	4704.45	< .001
Verbal	-0.53	-8.60	4594.26	< .001
Gossip	0.03	0.48	4288.59	.632
WTR_{own} (person-centered)	-0.16	-6.51	1072.91	< .001
Physical × WTR_{own} (person-centered)	0.10	6.09	4641.87	< .001
Verbal × WTR_{own} (person-centered)	0.12	7.08	4436.94	< .001
Gossip × WTR_{own} (person-centered)	0.05	3.35	4029.86	.001
WTR_{own} (person-average)	-0.12	-7.03	2552.25	< .001
Physical × WTR_{own} (person-average)	0.12	6.66	4648.92	< .001
Verbal × WTR_{own} (person-average)	0.08	4.36	4576.79	< .001
Gossip × WTR_{own} (person-average)	-0.0002	-0.02	4329.35	.987

Results from a linear mixed model with punishment type, $F(3, 4427.47) = 112.76$, $p < .001$, WTR_{own} (person-centered: $F(1, 697.57) = 15.69$, $p < .001$; person-average: $F(1, 922.13) = 26.37$, $p < .001$), and the WTR_{own} × punishment type interaction (person-centered: $F(3, 4253.25) = 19.92$, $p < .001$; person-average: $F(3, 4433.33) = 21.65$, $p < .001$). The table shows parameter estimates from planned contrasts, without adjustments for multiple comparisons. All tests are two-sided. Social exclusion motivations are used as the reference category. Physical: *"I felt like physically intervening to stop the offender."*; Verbal: *"I felt like yelling at or arguing with the offender."*; Gossip: *"I felt like sharing negative information about the offender to others."*; Social exclusion: *"I felt like excluding the offender from my social interactions in the future."* The model controls for gender and the gender × punishment type interaction. Source data are provided as a Source Data file.

Supplementary Table 3. Moral wrongness of norm violations and endorsement of punishment motivations

	Estimate	*t*	*df*	*p*
Intercept	1.26	6.38	3519.08	< .001
Physical	0.14	0.59	5838.62	.552
Verbal	0.02	0.09	5551.19	.930
Gossip	0.45	2.02	3532.99	.043
Moral wrongness (person-centered)	0.51	10.88	3217.97	< .001
Physical × Moral wrongness (person-centered)	-0.13	-2.71	4261.51	.007
Verbal × Moral wrongness (person-centered)	-0.14	-3.06	4386.70	.002
Gossip × Moral wrongness (person-centered)	-0.12	-2.58	3796.70	.010
Moral wrongness (person-average)	0.42	7.85	3542.27	< .001
Physical × Moral wrongness (person-average)	-0.22	-3.50	5839.16	< .001
Verbal × Moral wrongness (person-average)	-0.10	-1.57	5547.89	.116
Gossip × Moral wrongness (person-average)	-0.12	-1.94	3528.88	.052

Results from a linear mixed model with punishment type, $F(3, 4215.43) = 1.78$, $p = .148$, moral wrongness (person-centered: $F(1, 1471.93) = 120.06$, $p < .001$; person-average: $F(1, 1306.88) = 62.39$, $p < .001$) and the moral wrongness × punishment type interaction, (person-centered: $F(3, 4008.72) = 3.92$, $p = .008$; person-average: $F(3, 4213.49) = 4.19$, $p = .006$), as predictors of punishment motivations. The table shows parameter estimates from planned contrasts, without adjustments for multiple comparisons. All tests are two-sided. Social exclusion motivations are used as the reference category. Physical: *"I felt like physically intervening to stop the offender."*; Verbal: *"I felt like yelling at or arguing with the offender."*; Gossip: *"I felt like sharing negative information about the offender to others."*; Social exclusion: *"I felt like excluding the offender from my social interactions in the future."* The model controls for gender and the gender × punishment type interaction. Source data are provided as a Source Data file.

Supplementary Table 4. Relative power and endorsement of punishment motivations

	Estimate	*t*	*df*	*p*
Intercept	3.25	26.13	3733.10	< .001
Physical	-0.95	-6.50	5828.31	< .001
Verbal	-0.54	-3.72	5162.47	< .001
Gossip	0.12	0.89	3411.62	.376
Power (person-centered)	-0.16	-3.12	3594.75	.002
Physical × Power (person-centered)	0.06	1.20	4637.48	.228
Verbal × Power (person-centered)	0.13	2.53	4717.07	.011
Gossip × Power (person-centered)	0.01	0.14	3936.69	.885
Power (person-average)	-0.26	-4.09	3725.88	< .001
Physical × Power (person-average)	0.16	2.14	5822.19	.032
Verbal × Power (person-average)	0.11	1.50	5189.88	.132
Gossip × Power (person-average)	-0.05	-0.73	3461.04	.467

Results from a linear mixed model with punishment type, $F(3, 4066.10) = 22.51$, $p < .001$, power (person-centered: $F(1, 1635.80) = 7.18$, $p = .007$; person-average: $F(1, 1292.24) = 18.51$, $p < .001$), and the power × punishment type interaction (person-centered: $F(3, 4247.63) = 2.75$, $p = .041$; person-average: $F(3, 4114.19) = 3.36$, $p = .018$), as predictors of punishment motivations. The table shows parameter estimates from planned contrasts, without adjustments for multiple comparisons. All tests were two-sided. Social exclusion motivations are used as the reference category. Physical: *"I felt like physically intervening to stop the offender."*; Verbal: *"I felt like yelling at or arguing with the offender."*; Gossip: *"I felt like sharing negative information about the offender to others."*; Social exclusion: *"I felt like excluding the offender from my social interactions in the future."* The model controls for gender and the gender × punishment type interaction. Source data are provided as a Source Data file.

Supplementary Table 5. Anger, disgust, and endorsement of punishment motivations

	Estimate	*t*	*df*	*p*
Intercept	1.22	6.86	2814.86	< .001
Verbal	-0.78	-3.62	4292.11	< .001
Gossip	0.04	0.22	3679.08	.824
Social exclusion	-0.35	-1.70	4145.23	.088
Anger (person-centered)	0.19	4.79	970.36	< .001
Verbal × Anger (person-centered)	0.09	1.97	4756.23	.049
Gossip × Anger (person-centered)	0.05	1.05	4605.54	.293
Social exclusion × Anger (person-centered)	0.10	2.31	3992.02	.021
Anger (person-average)	0.19	3.56	3290.62	< .001
Verbal × Anger (person-average)	0.15	2.25	4665.96	.024
Gossip × Anger (person-average)	-0.03	-0.48	4092.95	.631
Social exclusion × Anger (person-average)	0.06	0.91	4324.04	.361
Disgust (person-centered)	0.12	3.26	1175.49	.001
Verbal × Disgust (person-centered)	0.03	0.70	4633.02	.483
Gossip × Disgust (person-centered)	0.11	2.75	4533.95	.006
Social exclusion × Disgust (person-centered)	0.10	2.61	3873.57	.009
Disgust (person-average)	0.05	1.06	4108.44	.289
Verbal × Disgust (person-average)	0.17	2.75	4923.72	.006
Gossip × Disgust (person-average)	0.22	3.76	4427.91	< .001
Social exclusion × Disgust (person-average)	0.24	4.07	4378.78	< .001

Results from a linear mixed model with punishment type, $F(3, 4345.83) = 6.23$, $p < .001$, anger (person-centered: $F(1, 372.55) = 67.16$, $p < .001$; person-average: $F(1, 1309.93) = 36.45$, $p < .001$), disgust (person-centered: $F(1, 450.90) = 42.18$, $p < .001$; person-average: $F(1, 1429.56) = 35.78$, $p < .001$), and the anger × punishment type (person-centered: $F(3, 4277.69) = 2.17$, $p = .089$; person-average: $F(3, 4262.32) = 3.04$, $p = .028$), and disgust × punishment type (person-centered: $F(3, 4166.67) = 3.79$, $p = .010$; person-average: $F(3, 4439.01) = 6.81$, $p < .001$, interactions as predictors of punishment motivations. The table shows parameter estimates from planned contrasts, without adjustments for multiple comparisons. All tests are two-sided. Physical confrontation motivations are used as the reference category. Physical: *"I felt like physically intervening to stop the offender."*; Verbal: *"I felt like yelling at or arguing with the offender."*; Gossip: *"I felt like sharing negative information about the offender to others."*; Social exclusion: *"I felt like excluding the offender from my social interactions in the future."*

The model controls for gender and the gender × punishment type interaction. Source data are provided as a Source Data file.
