# Supplementary material for: Direct and indirect punishment of norm violations in daily life
Source: Nat Commun. 2020 Jul 9;11:3432. doi: 10.1038/s41467-020-17286-2 (PMC7347610; doi:10.1038/s41467-020-17286-2)
Supplement: Supplementary file 3 — Reporting Summary [file 41467_2020_17286_MOESM3_ESM.pdf]

nature research

Corresponding author(s): Catherine Molho

Last updated by author(s): Jun 11, 2020

Reporting Summary

Nature Research wishes to improve the reproducibility of the work that we publish. This form provides structure for consistency and transparency in reporting. For further information on Nature Research policies, see Authors & Referees and the Editorial Policy Checklist.

Statistics

For all statistical analyses, confirm that the following items are present in the figure legend, table legend, main text, or Methods section.

n/a	Confirmed	
☐	☒	The exact sample size (*n*) for each experimental group/condition, given as a discrete number and unit of measurement
☐	☒	A statement on whether measurements were taken from distinct samples or whether the same sample was measured repeatedly
☐	☒	The statistical test(s) used AND whether they are one- or two-sided<br>*Only common tests should be described solely by name; describe more complex techniques in the Methods section.*
☐	☒	A description of all covariates tested
☐	☒	A description of any assumptions or corrections, such as tests of normality and adjustment for multiple comparisons
☐	☒	A full description of the statistical parameters including central tendency (e.g. means) or other basic estimates (e.g. regression coefficient) AND variation (e.g. standard deviation) or associated estimates of uncertainty (e.g. confidence intervals)
☐	☒	For null hypothesis testing, the test statistic (e.g. *F*, *t*, *r*) with confidence intervals, effect sizes, degrees of freedom and *P* value noted<br>*Give P values as exact values whenever suitable.*
☒	☐	For Bayesian analysis, information on the choice of priors and Markov chain Monte Carlo settings
☐	☒	For hierarchical and complex designs, identification of the appropriate level for tests and full reporting of outcomes
☐	☒	Estimates of effect sizes (e.g. Cohen's *d*, Pearson's *r*), indicating how they were calculated

Our web collection on statistics for biologists contains articles on many of the points above.

Software and code

Policy information about availability of computer code

Data collection	To collect data, we used the following software: Qualtrics (version June, 2018) to collect survey responses and SurveySignal (Hofmann & Patel, 2015) to contact participants via messages on their mobile phones.
Data analysis	To analyze the data, we used R (version 3.6.0) and SPSS (version 25). The SPSS syntax used for our main analyses is provided on the Open Science Framework: https://osf.io/du7mp/

For manuscripts utilizing custom algorithms or software that are central to the research but not yet described in published literature, software must be made available to editors/reviewers. We strongly encourage code deposition in a community repository (e.g. GitHub). See the Nature Research guidelines for submitting code & software for further information.

Data

Policy information about availability of data

All manuscripts must include a data availability statement. This statement should provide the following information, where applicable:

- Accession codes, unique identifiers, or web links for publicly available datasets
- A list of figures that have associated raw data
- A description of any restrictions on data availability

Data availability statement: All data that are relevant to the analyses described herein are available on the Open Science Framework (https://osf.io/du7mp/). The source data underlying Figs 1a-c, Tables 1-4, and Supplementary Tables 1-5 are provided as a Source Data file. A reporting summary for this Article is available as a Supplementary Information file.

Field-specific reporting

Please select the one below that is the best fit for your research. If you are not sure, read the appropriate sections before making your selection.

☐ Life sciences ☒ Behavioural & social sciences ☐ Ecological, evolutionary & environmental sciences

For a reference copy of the document with all sections, see nature.com/documents/nr-reporting-summary-flat.pdf

Behavioural & social sciences study design

All studies must disclose on these points even when the disclosure is negative.

Study description	This study has two main goals: (1) to document the extent to which people use different types of punishment - including direct confrontation, gossip, and social exclusion - in response to norm violations they have experienced or witnessed in daily life, and (2) to test hypotheses regarding the relations of multiple relational (i.e., valuation of offenders and identity of the victims), situational (i.e., severity of norm violation, and power), and emotional (i.e., anger and disgust) factors with different types of punishment. To accomplish these goals, the study relies on a longitudinal methodology (i.e., daily diary methodology), in which participants receive one questionnaire per day for two consecutive weeks (repeated measurements) via their mobile phones. The questionnaires are used to obtain descriptions of norm violations that participants encounter in natural settings, various types of contextual information, and individuals' motivations to punish offenders as well as their actual punishment behaviors.
Research sample	The sample consists of 257 participants recruited in the Netherlands. In this sample, 66.1% of participants identified as female (compare to a 33.27% documented as female in the Dutch 2018 census; source: Centraal Bureau voor de Statistiek); one participant chose the “other” option. Mean age was 39.15 years (SD = 16.02, range: 18-75 years). 34.6% of participants belonged to the 20-29 age group (compare to 12.6% in the Dutch population); 19.8% belonged to the 30-39 age group (12.1% in the Dutch population); 9.7% belonged to the 40-49 age group (13.4% in the Dutch population); 15.6% belonged to the 50-59 age group (14.5% in the Dutch population); 11.7% to the 60-69 age group (12.1% in the Dutch population); and 3.5% to the 70-79 age group (8.5% in the Dutch population). Thus, we obtained a good representation of different age groups, albeit skewed towards including younger participants and more female participants. Most participants (90.2%) were born in the Netherlands (compare to 93.94% in the census data), and were highly educated (60.5% had a bachelor’s or higher degree; 39.5% had secondary education, including vocational training). The average subjective socioeconomic status (measured via a 1 to 10-point ladder method) was above the scale midpoint (M = 6.49, SD = 1.54). The country of data collection was chosen based on convenience. The sample (and recruitment methods; see below) was chosen with the goal of increasing generalizability of findings (e.g., compared to using a student sample).
Sampling strategy	Two panel agencies were used to recruit a relatively diverse sample of individuals. One panel agency had access to a representative sample of the Dutch population; the other agency recruited via social media and forums. Thus, the sampling procedure involved a combination of random sampling and convenience sampling. The targeted sample size was pre-registered (pre-registration is available on the OSF: https://osf.io/fdzxt) and determined based on (a) considerations of good practices for studies using similar methodologies (i.e., daily diaries) and (b) financial and other practical constraints. We did not conduct a formal power analysis. Power analyses for linear mixed models are based on simulation and require setting a number of parameters that quickly increases with model complexity. In the absence of reliable information on these parameters, we decided against a formal power analysis. The final sample size is considered sufficient based on standards and recommendations in the literature (for examples, see references below).<br><br>Conner, T. S., & Mehl, M. R. (2011). Handbook of research methods for studying daily life. New York, NY: Guilford Press.<br>Smith, P. K., & Hofmann, W. (2016). Power in everyday life. Proceedings of the National Academy of Sciences of the United States of America, 113, 10043–10048. http://dx.doi.org/10.1073/pnas.1604820113<br>Righetti, F., Gere, J., Hofmann, W., Visserman, M. L., & van Lange, P. A. M. (2016). The burden of empathy: Partners’ responses to divergence of interests in daily life. Emotion, 16, 684–690. http://dx.doi.org/ 10.1037/emo0000163
Data collection	Data were collected (a) via computers placed in individual, separated cubicles in the lab of the VU Amsterdam and (b) via participants' mobile phones. No one else besides the participants (and the experimenter during the intake) was present during data collection. The first author conducted all intake sessions.
Timing	Data were collected between June and August 2018. There were no gaps within this data collection period.
Data exclusions	We did not exclude any participants from the analyses, but we only analyzed data from completed daily assessments and follow-up surveys. In some cases, due to technical reasons, participants were able to complete the same daily assessment or follow-up survey more than once. When they did so, we retained the first response. This was either the most complete response or, in few cases, one of multiple incomplete responses.
Non-participation	The response rate for daily assessments was 80.27%. The response rate for the first follow-up was 71.98%; the response rate for the second follow-up (40.02%) was lower. Due to a technical problem with the software used to automatically send surveys, 55 participants did not receive the second follow-up. When considering only participants who received the survey, the response rate for the second follow-up (62.38%) was higher.
Randomization	Participants were not allocated into experimental groups.

Reporting for specific materials, systems and methods

We require information from authors about some types of materials, experimental systems and methods used in many studies. Here, indicate whether each material, system or method listed is relevant to your study. If you are not sure if a list item applies to your research, read the appropriate section before selecting a response.

Materials & experimental systems

n/a	Involved in the study
☒	☐ Antibodies
☒	☐ Eukaryotic cell lines
☒	☐ Palaeontology
☒	☐ Animals and other organisms
☐	☒ Human research participants
☒	☐ Clinical data

Methods

n/a	Involved in the study
☒	☐ ChIP-seq
☒	☐ Flow cytometry
☒	☐ MRI-based neuroimaging

Human research participants

Policy information about studies involving human research participants

Population characteristics

See ' Research Sample' description above.

Recruitment

For details, see 'Sampling Strategy' section above. Two panel agencies were used to recruit participants. One panel agency had access to a representative sample of the Dutch population; the other agency recruited via social media and forums. Given the nature of this recruitment procedure, self-selection bias is possible. This bias could affect conclusions about the prevalence rates of norm violations in daily settings. As we have noted in our discussion, the prospective data collection method we have employed could have made participants more attentive to norm violations occurring in daily settings, while also increasing the overall frequency of their interventions. This might have been particularly the case in a sample that self-selected to participate in this type of research. That said, we believe this issue does not affect our conclusions regarding how relational, situational, and emotional factors relate to the use of direct versus indirect strategies of punishment in response to the reported offenses.

Ethics oversight

This research was approved by the Research Ethics Committee of the VU Amsterdam (#VCWE-2018-052).

Note that full information on the approval of the study protocol must also be provided in the manuscript.
